# Application of antigen presenting cell-targeted nanovaccine delivery system in rhabdovirus disease prophylactics using fish as a model organism

**DOI:** 10.1186/s12951-020-0584-x

**Published:** 2020-01-30

**Authors:** Chen Zhang, Gao-Xue Wang, Bin Zhu

**Affiliations:** 1 College of Animal Science and Technology, Northwest A&F University, Yangling, 712100 China; 2 Northwest A&F University, Xinong Road 22nd, Yangling, Shaanxi 712100 China

**Keywords:** Targeted nanovaccine, Mannose, Carbon nanotubes, Rhabdovirus, Immune response

## Abstract

**Background:**

Targeted delivery of virus-associated antigens to professional antigen-presenting cells (APCs) is considered as an efficient strategy to enhance the pyrophytic effect of vaccines against rhabdovirus disease.

**Materials and methods:**

In this study, we constructed a targeted carbon nanotubes-based vaccine deliver system (SWCNTs-MG) which can recognize the signature receptor (mannose) of APCs. An environmentally and economically important disease called spring viremia of carp (SVC) was studied as a model to evaluate the feasibility of single-walled carbon nanotubes (SWCNTs) conjugated with mannosylated antigen for rhabdovirus prevention.

**Results:**

Results showed that SWCNTs-MG could cross into fish body and present to internal immune-related tissues through gill, muscle and intestine within 6 h immersed vaccination. With further modification of mannose moiety, the obtained nanovaccine showed enhanced uptake by carp macrophages and immune-related tissues, which would then trigger strong immune responses against spring viremia of carp virus (SVCV) infection. Moreover, the survival rate of fish vaccinated with SWCNTs-MG (30 mg/L) was 63.5% after SVCV infection, whereas it was 0% for the control group.

**Conclusion:**

This study not only provide a theoretical basis and research template for the application of targeted nanovaccine system in aquatic animals, but also play an important role in supporting development of healthy aquaculture and ensuring the safety of aquatic products and ecology.
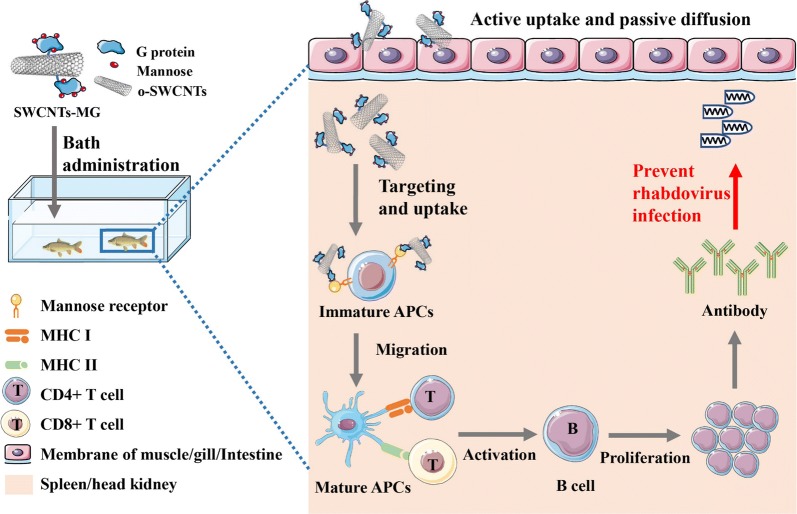

## Introduction

Rhabdoviridae-related viruses are a kind of viruses with negative sense single strained RNA and a variety of hosts [1, 2]. Diseases caused by rhabdovirus pose a serious threat to most of the vertebrates [3–5]. Prophylactic vaccine is considered as the most effective measure to prevent rhabdovirus infection [6, 7]. However, due to the biological barrier (such as skin, selective permeability of the cell membrane, gastrointestinal tract and so on), it is not easy for most biological macromolecules including antigen proteins and plasmid enter into host and play a role, which led to the less robust immune responses of current rhabdovirus vaccines [8–10]. Therefore, developments in efficient delivery technologies for vaccine play a vital role to prevent rhabdovirus diseases.

An effective nanovaccine delivery system is commonly composed of the antigens, delivery carrier, and adjuvant [11, 12]. For most rhabdovirus, the surface glycoprotein (G) of rhabdovirus is considered as a major antigen that could induce a primary host immune response. As the typical rhabdovirus, SVCV G protein is the most commonly protein used in SVCV vaccine constructs [13]. As a promising carrier, single-walled carbon nanotubes (SWCNTs) has been widely used for antigens and drugs delivery attributable to its excellent properties, such as biocompatibility, needle-like structure, and high carrying capacity [14–16]. Specifically, SWCNTs are uniquely equipped to deliver cargos (such as antigens and drugs) across biological membranes [17], their use for vaccination could allow effective utilization of antigens that have previously not been able to induce adequate or appropriate responses, as well as providing significant means of enhancing and modulating immune response [18]. In order to further enhance the efficacy of vaccination, the adjuvants are essential components and usually co-administrated with immunogens, especially for the weak immunogens [19]. As the efficient adjuvant and the targeted ligands which can specifically recognize the signature receptor (mannose) on antigen-presenting cells (APCs) such as macrophages and dendritic cells, mannose has been widely used for the construction of targeted nanovaccine [20, 21]. Therefore, mannose was modified and conjugated to antigens by chemical synthesis in this study.

To date, targeted delivery has been widely used in cancer treatment, with few studies focusing on the prevention of viral disease especially the rhabdovirus. In this study, spring viremia of carp virus (SVCV) was studied as a model to evaluate the feasibility of targeted nanovaccine in preventing rhabdovirus diseases. SVCV is a cytopathic virus belonging to the genus *Vesiculorius* of the family *Rhabdoviridae* [22]. The notable advantages of employing SVCV as the model including the followings: (1) Safety, aquatic animals constitute the narrow nature host range of SVCV, and humans are nonsusceptible. (2) Representative to rhabdovirus, SVCV is a typical rhabdovirus with its genome composed of a negative, single-stranded RNA. (3) Widespread distribution and easy accessibility, SVCV has been reported in worldwide and is susceptibility to almost cyprinid [22–25].

In this study, a targeted delivery system based on SWCNTs conjugated to mannosylated antigens was constructed. The targeting ability and uptake kinetics of the targeted delivery system was checked both in vivo and in vitro. Moreover, for demonstrating the targeted delivery system could act as an effective platform for prophylactic vaccines against rhabdovirus disease, the immune responses in vaccinated fish were evaluated. This work highlights the great potential of SWCNTs-based targeted vaccine delivery system as an attractive platform to prevent rhabdoviral diseases.

## Results and discussion

### Construction and characterization of targeted delivery system

**Fig. 1:**
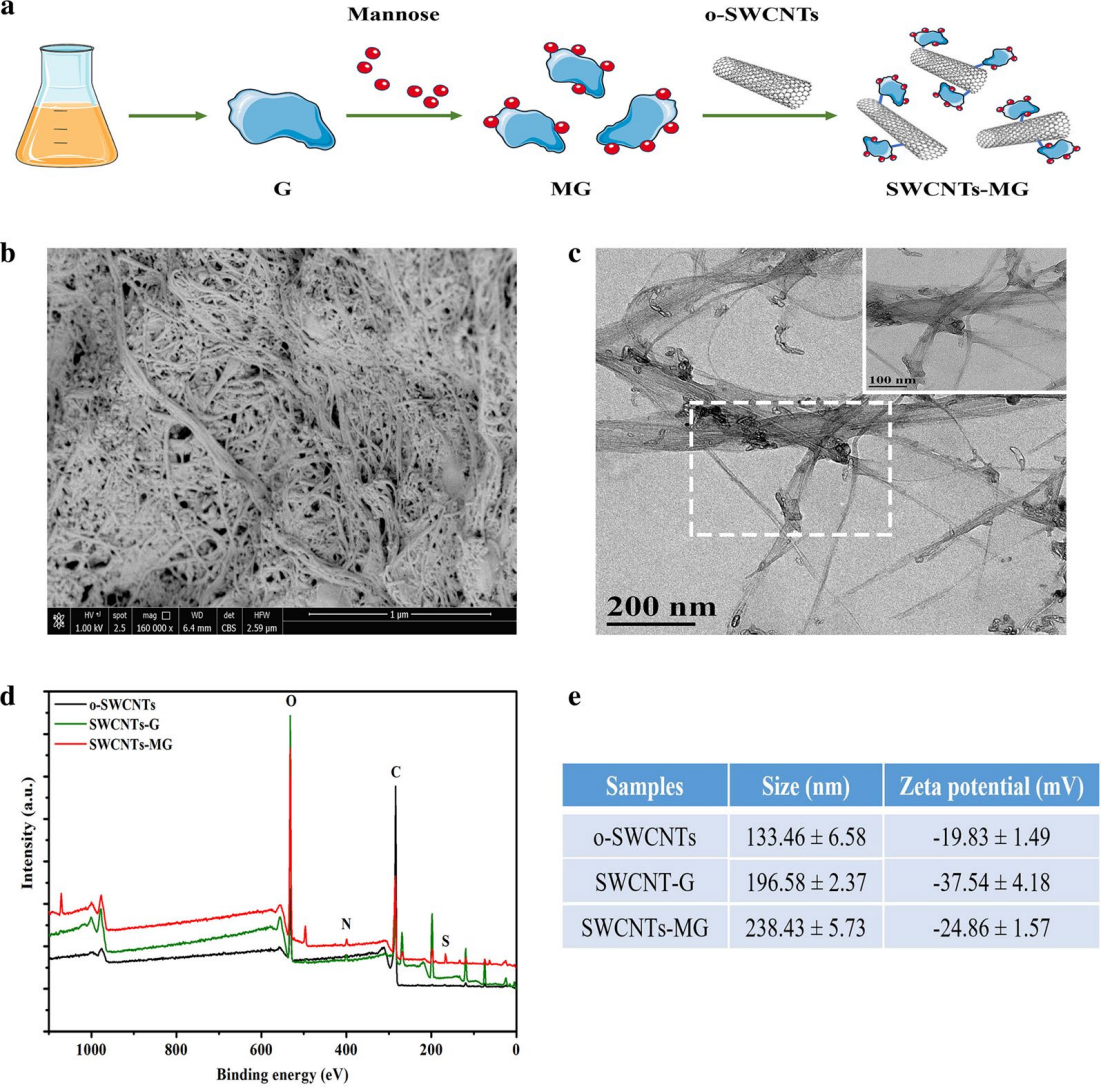
Characterization of nanovaccine. **a** Schematic illustration to show the step-by-step preparation of SWCNTs-MG nanovaccine. **b** Representative scanning electron microscopy image and **c** transmission electron microscopy image of SWCNTs-MG nanovaccine. **d** X-ray photoelectron spectroscopy analysis. **e** Particle size and zeta potential analysis

Antigen, adjuvant, and delivery carrier are the key elements for effective nanovaccine delivery system. As illustrated in Fig. 1a, SVCV antigen protein (G) were modified with mannose, and then encapsulated with SWCNTs to construct the targeted nanovaccine delivery system (SWCNTs-MG). Furthermore, the obtained SWCNTs-MG nanovaccine was characterized. As revealed by scanning electron microscopy (SEM) and transmission electron microscopy (TEM), the constructed nanovaccine is a tubular structure with its surface conjugated with mannosylated antigen proteins (Fig. 1b, c). Further confirmation of the synthetic constructs was performed using X-ray photoelectron spectroscopy (XPS) spectrum. The XPS spectrum of SWCNTs-MG shows two characteristic peaks of SWCNTs (carbon (288 eV) and oxygen (532 eV)) (Fig. 1d). The particle size and zeta potential of the vaccines were analyzed. As show in Fig. 1e, the average sizes of o-SWCNTs, SWCNTs-G, and SWCNTs-MG were 133.46 nm, 196.58 nm, and 238.43 nm, respectively. Upon conjugated with mannosylated antigens, the resulting SWCNTs showed increased size to be about 105 nm. Moreover, zeta potential revealed a negative surface charge (− 19.83 ± 1.49 mV) for SWCNTs which decrease to − 24.86 ± 1.57 mV after the conjugation of mannosylated antigens. Moreover, as measured by bicinchoninic acid (BCA) protein assay and phenol–sulfuric acid colorimetry, the SWCNTs-MG nanovacine containing 3.4% mannose and 40.2% antigen protein.

### Safety evaluation of SWCNTs-MG

**Fig. 2:**
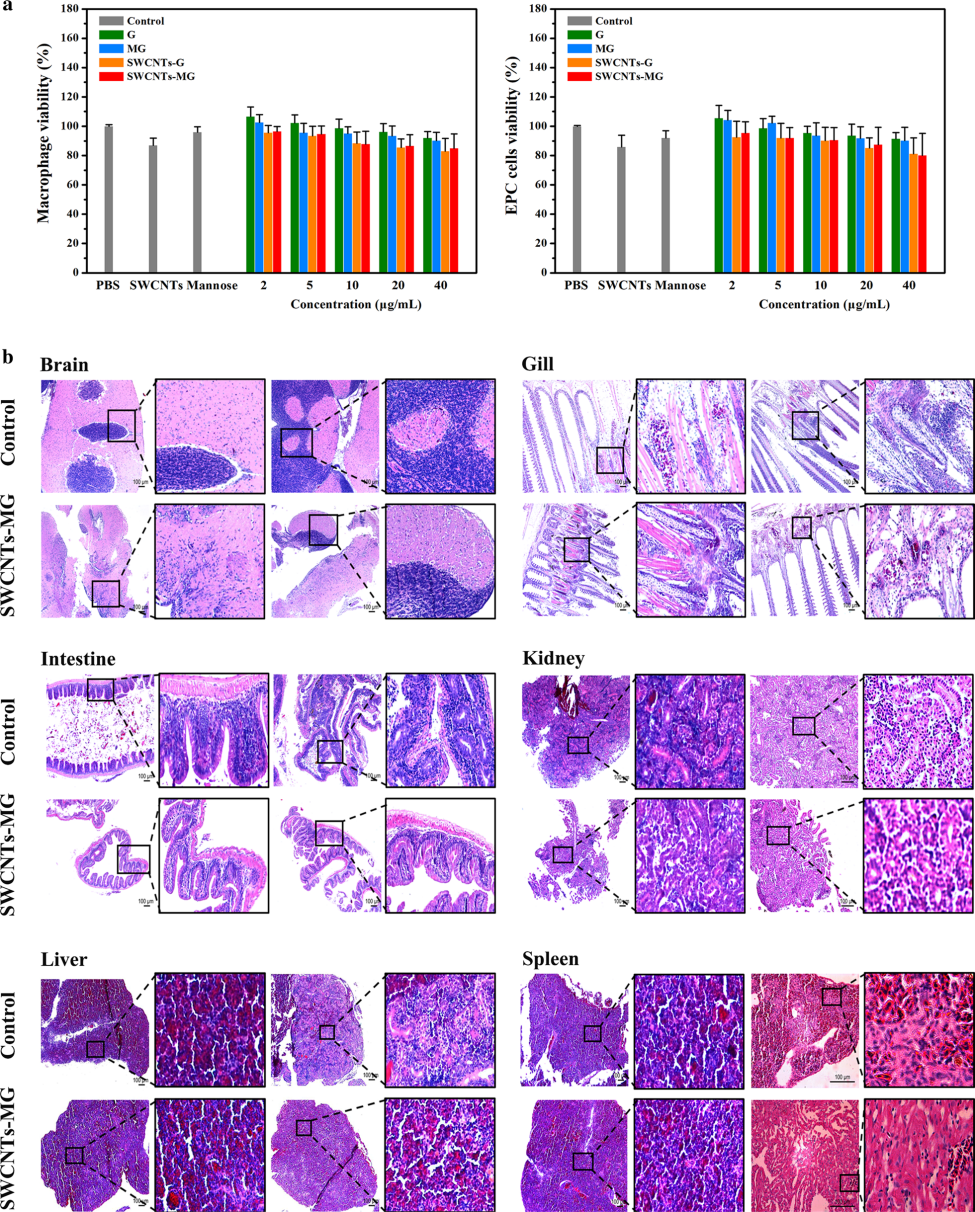
Safety evaluation of nanovaccine in vivo and in vitro. **a** Relative cell viability of carp macrophage and EPC cells after incubation with different concentrations of G, MG, SWCNTs-G and SWCNTs-MG for 24 h. **b** Histopathologic analyses of H&E-stained tissue sections from the brains, gills, intestines, kidneys, livers, and spleens of SWCNTs-MG vaccinated fish after immersed for 10 h, scale bar: 100 μm

Vaccine safety is first priority to be considered before vaccination. After the SWCNTs-MG nanovaccine was constructed, its safety was evaluated in vitro and in vivo. The potential cytotoxicity of SWCNTs-MG toward macrophages and EPC cells was determined by the cell viability assy. As Fig. 2a shown, after macrophages and EPC cells incubated with 40 μg/mL SWCNTs-MG for 24 h, the survival rate of both kinds of cells shown no significant difference with control groups. The safety evaluation was also performed in common carp, after common carp immersed with 60 mg/L SWCNTs-MG for 24 h. As depicted in Fig. 2b, no damage nor abnormality was found in vaccinated fish brain, gill, intestine, kidney, liver, and spleen. Besides, within 60 d after immersion immunization, there was no lesion nor abnormality in vaccinated carp when compared with control group. This study indicate SWCNTs-MG nanovaccine has good biocompatibility in vitro and in vivo.

Up to now, the safety of carbon nanotubes remains controversial. Some studies suggested that carbon nanotubes may be toxic: Warheit indicated that pulmonary exposures to SWCNT in rats produced a non-dose-dependent series of multifocal granulomas, which were evidence of a foreign tissue body reaction and were nonuniform in distribution and not progressive beyond 1-month postexposure [26]. Zhu conjectured that high concentration CNTs (above 100 mg/L) might induce toxicity in rare minnow (*Gobiocypris rarus*), in addition, o-SWCNTs (188.2 mg/L) could induce apoptosis in *S. cerevisiae* cells, and oxidative stress in activation of the mitochondria-dependent apoptotic pathway [27, 28]. However, no conclusive evidence could verify the toxicity of CNTs. Numerous studies indicated CNTs is biocompatibility: Rats were used as the model to analyze the toxic of SWCNTs, results showed that rat exposure to SWCNTs did not produce mortality, changes in clinical signs, or body weights during the observation period [29]. Dumortier indicated that functionalized carbon nanotubes are non-cytotoxic and preserve the functionality of primary immune cells [30]. In this study, we have purified and functionalized the SWCNTs, and then conjugated the SWCNTs with mannosylated antigen proteins. Studies suggested that when carbon nanotubes are purified and functionalized, their biological toxicity could be reduced [31–33]. Notably, after functionalized carbon nanotubes were further chemically modified with active substances (such as antigenic proteins), their biocompatibility is further enhanced and water-dispersibility is improved [34].

### Celluar uptake of nanovaccine by cyprinid macrophage

**Fig. 3:**
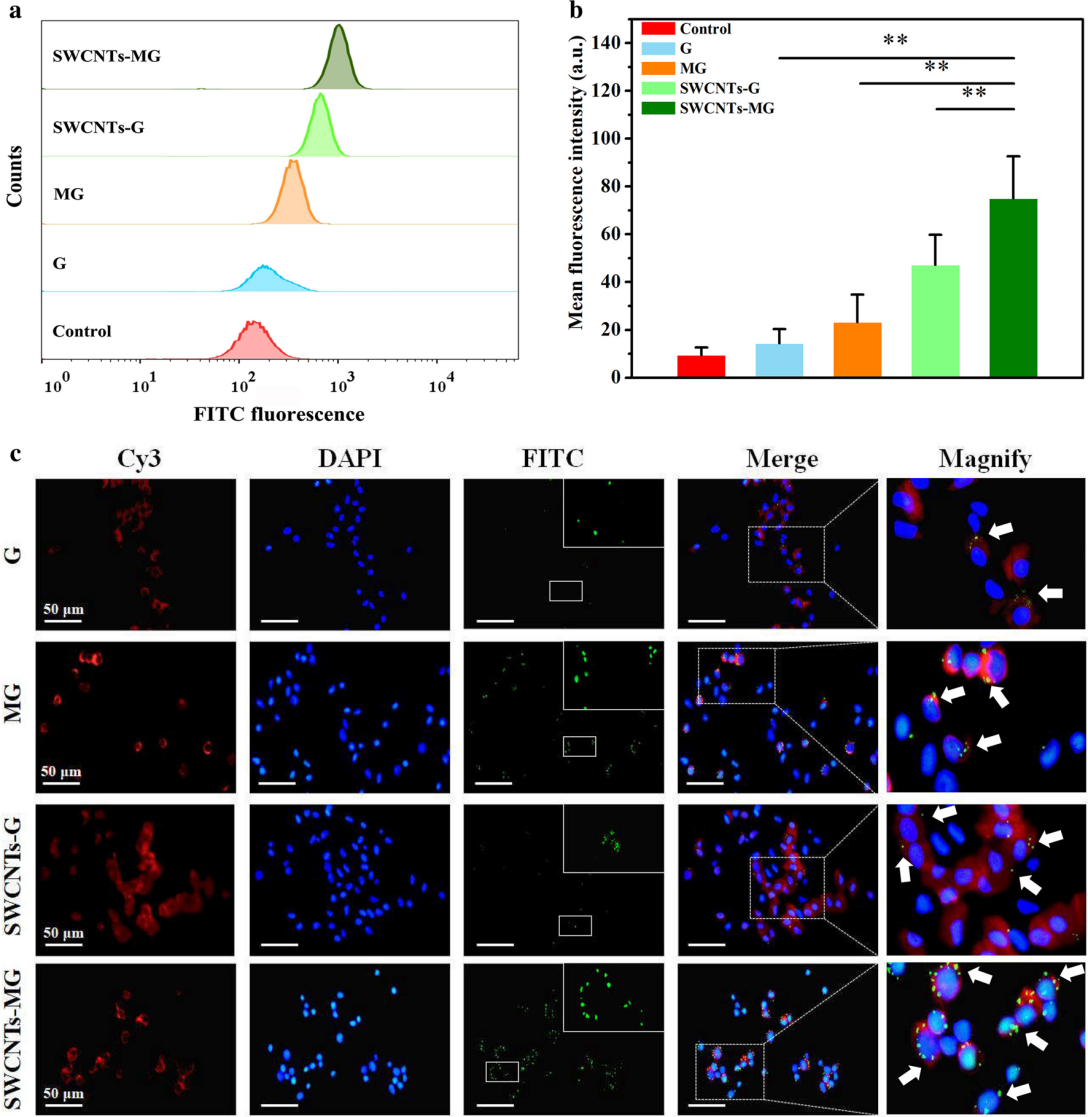
Cellular uptake of nanovaccine by carp macrophage in vitro. **a** Uptake of FITC labelled nannovaccines by carp macrophage in vitro. **b** Mean florescence intensity of cell uptake capability. **c** Representative confocal microscopic images of carp macrophage incubated with G, MG, SWCNTs-G, and SWCNTs-MG respectively. Vaccines were labeled with FITC (green channel, white arrows), respectively; The cell nucleus was labeled with DAPI (blue channel); The mannose receptor was labeled with Cy3 (red channel)

It is important for immune responses induced by vaccines that viral antigens are processed and presented by APCs [35]. Therefore, the cellular uptake of nanovaccine by APCs would be important for the efficacy of nanovaccines. To evaluate the cell uptake of nanovaccines by APCs, macrophages were incubated with different vaccines (G, MG, SWCNTs-G, and SWCNTs-MG) labeled with FITC for the flow cytometric analysis and immunofluorescence, respectively. As shown in Fig. 3a, b, SWCNTs-MG showed significantly enhanced cellular uptake by macrophages compared to SWCNTs-G (without mannose modification) (*P* < 0.01). Such a phenomenon was also confirmed by confocal fluorescence imaging of macrophages incubated with these nanovaccines (Fig. 3c). The enhanced cell uptake of nanovaccines containing antigen and adjuvant by APCs would be greatly favorable for inducing stronger immune responses and more effective vaccination.

### Detection of nanovaccine in fish tissues

**Fig. 4:**
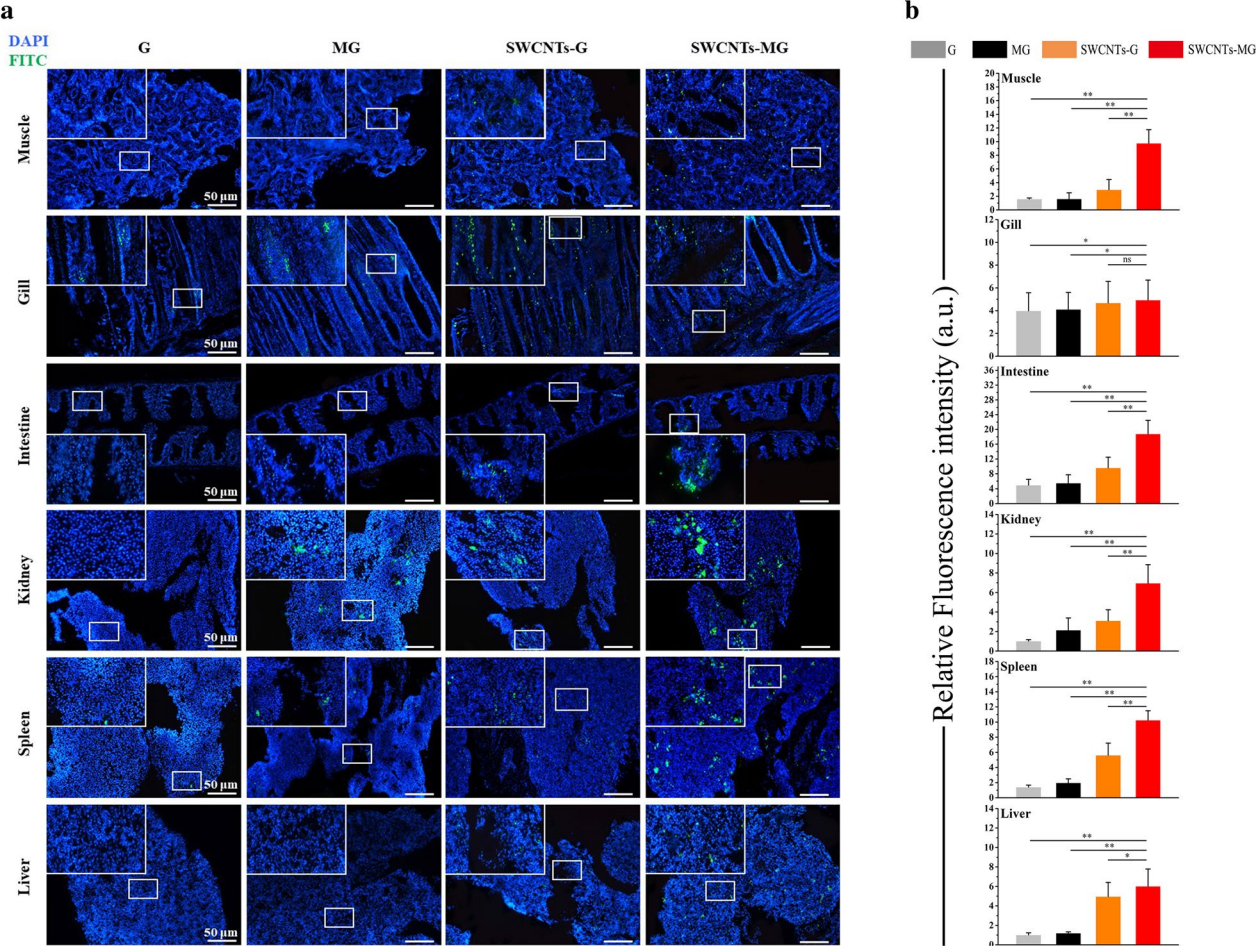
Uptake of nanovaccine in fish tissues. **a** The immunofluorescence images of carp tissues (gill, intestine, kidney, spleen, and liver) after incubated with vaccines (G, MG, SWCNTs-G, and SWCNTs-MG) respectively. **b** Mean fluorescence intensity of vaccine system in fish tissues. The vaccines were labeled with FITC (green channel); the cell nucleus was labeled with DAPI (blue channel). Data are presented as the mean ± SD. **P < 0.01, *P < 0.05

Due to skin barrier and selective permeability of the cell membrane, it is not easy for most biological macromolecules including proteins, drugs, and plasmid enter into fish body, which is also the obstacle for vaccine applications [36]. Moreover, the amount of vaccines enter into immune tissues would greatly determine the quality of induced immunities [37]. To tackle the obstacle of vaccines entering into host, herein SWCNTs was used as the vaccine carrier. As a promising vaccine carrier, SWCNTs possess numerous properties including penetrability, high carrying capacity, biocompatibility and so on [38]. As shown in Fig. 4, the content of SWCNTs-MG nanovaccines in vaccinated fish muscle, intestine, kidney, spleen, and liver were significantly higher than other vaccines (G, MG, and SWCNTs-G) respectively (*P *< 0.01) with stronger green fluoresces. These results suggest that the constructed nannovaccine delivery system, using SWCNTs as carrier and mannose as targeting ligand/adjuvant, could efficiently delivery nanovaccine into the immune related tissues via bath administration. With more antigens enter into immune related tissues, higher immune response would be induced.

### The maturation of APCs induced by nanovaccines

**Fig. 5:**
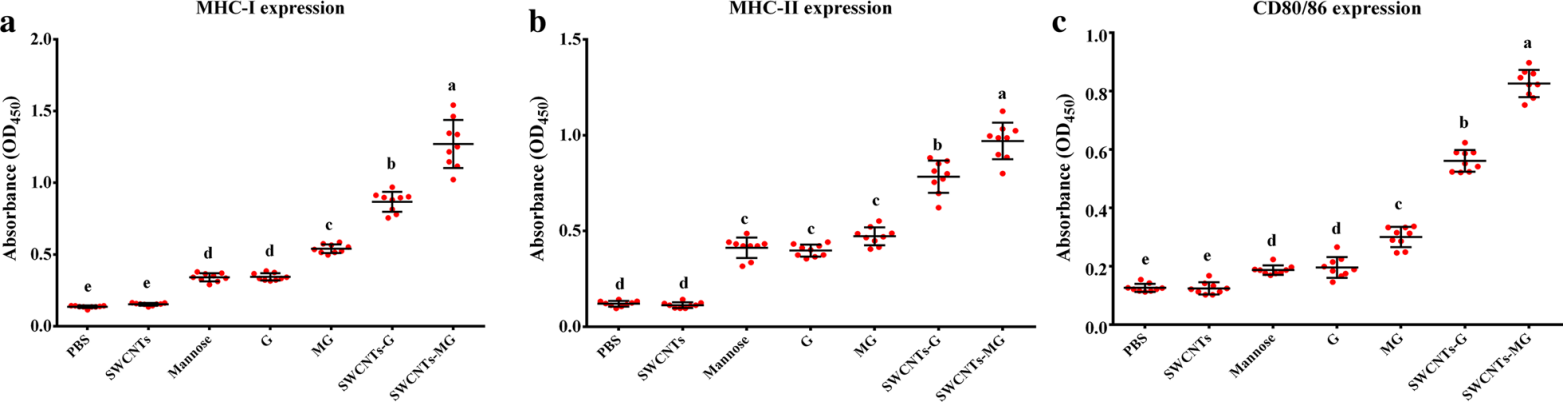
Effect of nanovaccines on antigen presenting cells maturation and antigen presentation. Data are represented as mean ± SD (n = 9). Data at the same sampling time with different letters are significantly different (*P* < 0.05)

After the immature APCs captured antigens, it could be stimulated into a matured status, which could lead to antigen presentation and then activate T cells with the subsequent immune response induced. As the surface markers for mature APCs, the expression level of MHC-I, MHC-II, and CD80/86 could reflect the maturation of APCs. Therefore, to evaluate the abilities of these nanovaccines to stimulate APCs maturation and antigen presentation, the expression of MHC-I, MHC-II, and CD80/86 in vaccinated fish were analyzed by ELISA. As depicted in Fig. 5, compared to samples treated with PBS and SWCNTs, those vaccinated with mannose, G, MG, SWCNTs-G, and SWCNTs-MG showed significantly increased secretion levels of MHC-I, MHC-II, and CD80/86, respectively. In addition, the highest secretion levels of these molecules were found in samples treated with SWCNTs-MG. Therefore, these data indicate that SWCNTs-MG nanovaccine appears to be the effective method for in vivo activation of APCs.

### The delivery kinetics of targeted nanovaccine

**Fig. 6:**
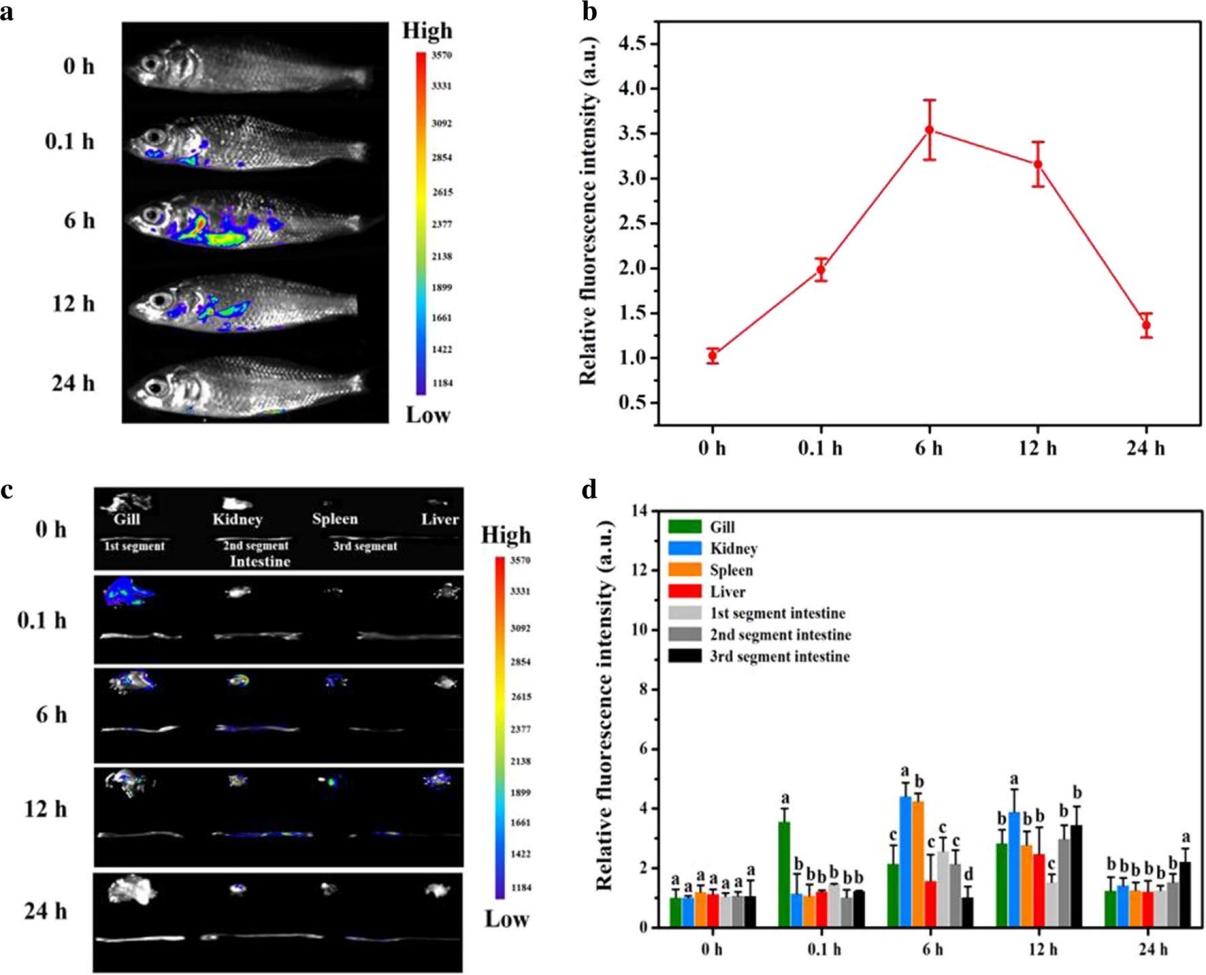
In vivo and ex vivo fluorescence images of vaccine system in vaccinated fish. **a** Representative in vivo fluorescence images of common carp at different time points after vaccination; **b** Quantitative fluorescence signals of vaccinated fish; **c** representative ex vivo fluorescence images of isolated fish tissues at different time points; **d** Quantitative fluorescence signals of different fish tissues. Data are means for three assays and represented as mean ± SD. Data at the same sampling time with different letters are significantly different (*P* < 0.05)

To investigate the delivery kinetics of targeted nanovaccine in vaccinated fish, common carp (1.0 ± 0.2 g) were exposed to SWCNTs-MG nanovaccine by immersion for 6 h and then transferred to standard dilution water. Fluorescence imaging was used to track SWCNTs-MG nanovaccine labeled with FITC. As shown in Fig. 6, from the beginning of immersed immunity at 0 h to the end of vaccination at 6 h, the content of SWCNTs-MG was gradually increased. Notably, the content of SWCNTs in kidney and spleen of vaccinated fish were significantly higher than that in other tissues (*P *< 0.05). Numerous studies indicated that kidney and spleen contain large amounts of macrophages [39]. The higher content of nanovaccine in these two immune organs reflecte the targeted delivery capacity of SWCNTs-MG nanovaccine, which showed that SWCNTs-MG could cross into fish body and present to internal immune-related tissues through gill, muscle and intestine within 6 h immersed vaccination. After the vaccinated fish transferred to standard dilution water, the intensity fluoresce of SWCNTs-MG nanovaccine was gradually decreased. Up to 24 h, the fluoresce was barely visible, which suggeste SWCNTs could excrete from the fish body. Zhu indicated that the CNTs could completely excreted out from the larvae at around 144 h [27], which is corresponding with our results. However, the more specific delivery kinetics of targeted nanovaccine needs further investigation. The current delivery kinetics of targeted nanovaccine suggests the targeted delivery ability and biocapacity of SWCNTs-MG nanovaccine.

### The immune response in vaccinated fish

**Fig. 7:**
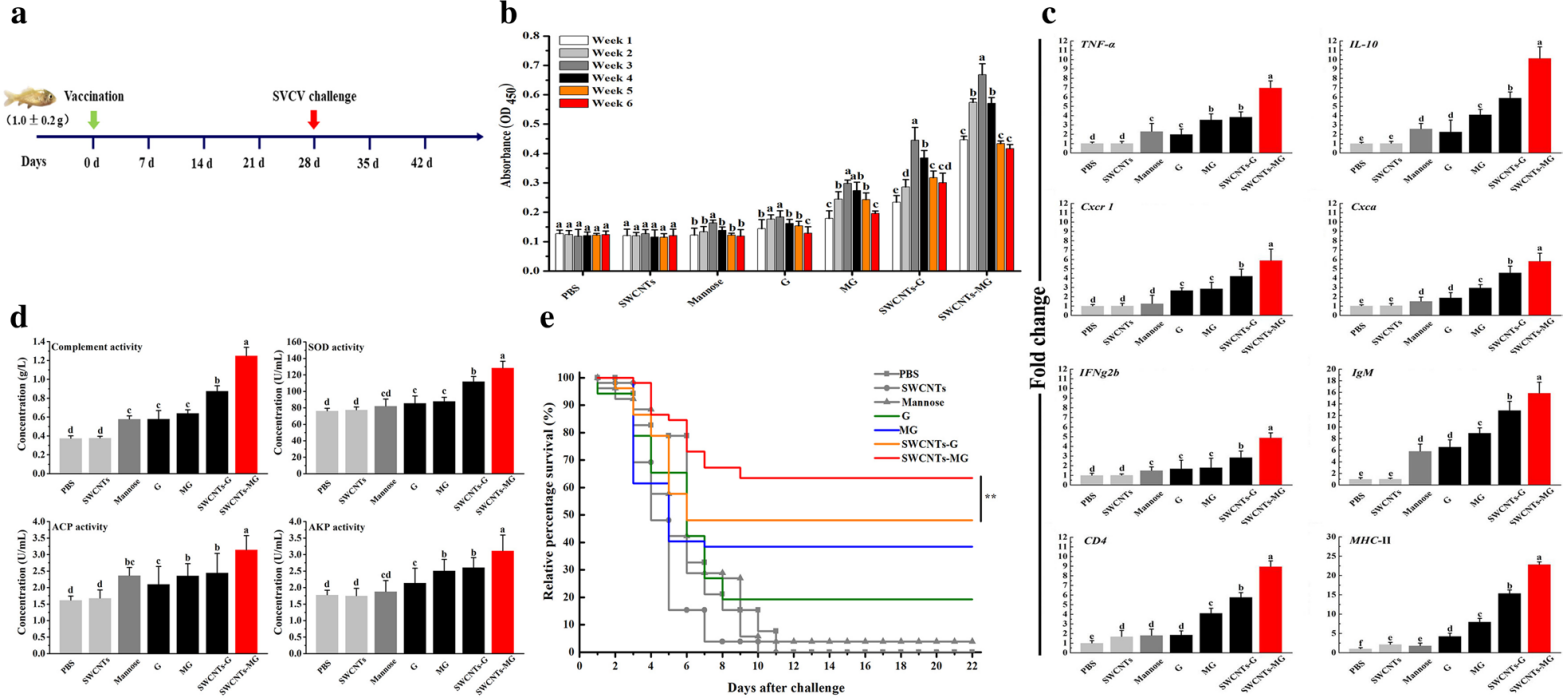
Immune response in vaccinated fish. **a** Schematic illustration to show the SVCV challenge experiment design. **b** Specific antibody levels of fish vaccinated with vaccines. **c** qRT-PCR analysis of the expression of immune genes in common carp via bath administration. **d** Enzyme activities in vaccinated common carp. Data are means for three assays and represented as mean ± SD. Data at the same sampling time with different letters are significantly different (*P* < 0.05). **e** Relative percentage survival after artificial challenging with SVCV in vaccinated common carp. The percentage survival was recorded daily and calculated at the end of the monitored period. *P* values were calculated by Log-rank (Mantel–Cox) Test (*P < 0.05, **P < 0.01)

To evaluate the prophylactic effects of targeted nanovaccine, we analyzed the immune response in vaccinated fish. As depicted in Fig. 7, SWCNTs-MG could induce higher levels of immune response including serum antibody production, enzyme activities, and immune genes expression than other vaccines (G, MG, and SWCNTs).

Results of the specific serum antibody and non-specific parameters reveal that eliciting powerful and long-lasting humoral or cellular immune response could be induced in vaccinated fish (Fig. 7b, d). Although production of antibodies does not necessarily correlate with protection and could vary with vaccine formulation, fish size, and environment [40], but to a certain extent, the antibody level could reveal the immune effect of vaccine [41, 42]. The enhancement of specific serum antibody response in vaccinated fish was prominent, in addition, the antibody level in SWCNTs-MG vaccinated fish were significantly higher than that in other vaccinated fish (G, MG, and SWCNTs-G) at the same dose. The significant enhancement of enzyme activities such as complement activities, superoxide dismutase (SOD), acid phosphatase (ACP), and alkaline phosphatase (AKP) activities were observed in SWCNTs-MG vaccinated fish. The complement system represents a major component innate immunity and also acts to enhance the adaptive immune response [43]. The improved complement activity in SWCNTs-MG vaccinated fish reveal the enhanced activation of complements pathway. As a vital antioxidant enzyme, SOD is an important factor for initiating the host immune response by regulating innate and acquired immunity [44]. ACP and AKP assays are the symbols of macrophage activation that reflect the ability of intracellular digestion of phagocytized antigens in the immune system [45].

To further investigate the defense mechanisms induced by the constructed SWCNTs-MG nanovaccine against SVCV infection, the expressions of immune-related genes were analyzed following vaccination. As shown in Fig. 7c, the immune-related genes including immunoglobulin (*IgM*), tumour necrosis factor alpha one (*TNF*-*α*), interleukin 10 (*IL*-*10*), CXC chemokine receptor-1 (*Cxcr 1*), CXC chemokine a (*Cxca*), interferon gamma-2beta (*IFNg2b*), cluster of differentiation 4 (*CD4*), and major histocompatibility complex class II (*MHC*-II) were significantly up-regulated in all vaccinated carps (*P* < 0.05). Importantly, the expressions of these immune-related genes in SWCNTs-MG vaccinated fish were significantly higher than other vaccines (G, MG, and SWCNTs-G) (*P* < 0.05). Interestingly, consistent with the increased production of specific serum antibodies, the expression of *IgM* gene was significantly up regulated in SWCNTs-MG vaccinated fish. *IgM* is a major component of the humoral immune system of teleost fish [46, 47]. The significantly enhancement of *IgM* in SWCNTs-MG treated fish indicate the enhanced immune response could be induced by SWCNTs-MG nanovaccine. Cytokines play a vital role in regulating host defense network [48, 49]. *TNF*-*α*, *IL*-*10*, *IFNg2b*, *Cxcr 1*, and *Cxca* are the important component of innate immunity [50–52]. The activation of innate immunity mediated by these cytokines could then condition the initiation of specific adaptive immune responses [53]. The expression of *CD4* and *MHC*-II are the typical markers reflecting the exogenous antigen presentation, the higher expression levels of *CD4* and *MHC*-II lead to increased advantages in terms of antigen presentation [54].

Specifically, SVCV challenge was used to further investigate the prophylactic effects of SWCNTs-MG nanovaccine. As depicted in Fig. 7e, the highest survival rate (63.5%) was observed in SWCNTs-MG vaccinated fish. Furthermore, as an adjuvant and targeted ligand, mannose can enhance 15.4% of the survival rate in SWCNTs-MG vaccinated fish compared with SWCNTs-G (without mannose) immunized fish.

Taken together, the significantly enhancement of immune response induced by SWCNTs-MG nanovaccine is possibly due to these following reasons: (1) SWCNTs as a promising carrier could pass through cell membranes and delivery more vaccine into the host, namely, SWCNTs make the constructed nanovaccine easier for attachment to specific target tissues and cells; (2) SWCNTs-MG could efficiently target APCs and then activate immune cells to induce strong immune responses; (3) mannose is an adjuvant that can be used to enhance immune response.

## Conclusions

In this study, we have successfully constructed a nanovaccine (SWCNTs-MG) composed of several key elements involving SWCNTs as the vaccine carrier, and mannose as an APCs-recognition moiety. SVCV was studied as a model to evaluate the feasibility of SWCNTs conjugated with mannosylated antigens against rhabdovirus infection. SWCNTs-MG could cross into fish body and present to internal immune-related tissues through gill, muscle and intestine within 6 h immersed vaccination. Moreover, mannose modification could facilitate the binding and cellular uptake of nanovaccine by APCs and further enhance host-protective immune responses against SVCV infection. Therefore, the study so far indicated that SWCNTs conjugated with mannosylated antigens are effective means against rhabdovirus infection. Importantly, this study shows a bright future for preventing rhabdovirus infection by using SWCNTs-based targeted nanovaccine delivery system.

## Materials and methods

### Virus and cell

SVCV (strain 0504) kindly provided by Professor Qiang Li (Dalian Ocean University, Dalian, China), was propagated in epithelioma papulosum cyprini (EPC) cells as previously described [55].

Carp macrophage was separated from common carp head kidney by using Fish tissue mononuclear cell separation kit (Solarbio, China). Epithelioma papulosum cyprinid (EPC) cells (kindly provided by Prof. Ling-bing Zeng in Yangtze River Fisheries Research Institute, Wuhan, Hubei, China) were cultured at 25 ± 0.5 °C in humidified atmosphere with 5% CO_2_, and maintained in Medium 199 (Hyclone, USA) supplemented with 10% fetal bovine serum (FBS; ZETA LIFE, USA).

### Animals

Common carps (*C. carpio*) weighing 1.0 ± 0.2 g were purchased from a local SVCV-free farm in Yangling (shannxi, China). Carps were bred in laboratory for 28 days prior to vaccination. The water temperature for common carps were maintained at 20–23 °C. Commercial dry feed pellets (Hellow Fish Dry Pellets; CVM Products, Beijing, China) were used to fed carps twice daily. All of the experimental animals were handled according to the guidelines of the Animal Experiment Committee, Northwest A&F University.

### Functionalized SWCNTs

Pristine SWCNTs purchased from Chendu Organic Chemicals Co., Ltd., Chinese Academy of Sciences (Chendu, China) were oxidized by H_2_SO_4_/HNO_3_ mixture (3:1, v/v) to form carboxyl groups on the surface of SWCNTs (o-SWCNTs) under reflux with stirring at room temperature for 48 h followed by our previous studies [24].

### Synthesis of functionalized mannose

We synthesized 1-(Isothiocyanates phenol)-2,3,4,6-O-α-d-glucopyranose (Additional file 1: Figure S1F) using well known protection group and coupling chemistry of glycosides. In the first step galactose or glucose were completely protected using acetanhydride in pyridine. Afterward the acetyl protected pyranose was treated with p-nitrophenol in boron trifluoride ethyl ether complex yielding the compound C (Additional file 1: Figure S1C). In the next step the intermediate was reacted with sodium methoxide yielding the compound D (Additional file 1: Figure S1D). Then compound D was hydrogenated by the Pd/C to get the compound E (Additional file 1: Figure S1E). Finally, the target compound F (Additional file 1: Figure S1F) was obtained by the reaction with CSCl_2_.

General ^1^H and ^13^C NMR spectra were measured with a Bruker AM500 spectrometer at 500.23 and 125.78 MHz. The chemical shifts are expressed in parts per million (δ value) downfield from tetramethylsilane,using tetramethylsilane (TMS) (δ = 0) and/or residual solvents such as Dimethyl sulfoxide (δ = 2.50) as an internal standard. Measurements of mass spectra were performed with an Electro spray-mass spectrometry mass spectrometer (Thermo Scientific™ LCQ Fleet™). Throughout this study, silica gel H (200–300 mesh; Qingdao Marine Chemical Factory, China) was used for the column chromatography. For TLC plates, Silica gel (GF_254_) (Qingdao Marine Chemical Factory, China) were used for thin layer chromatographic (TLC) analysis, and all of the spots and bands were detected by UV irradiation (254, 365 nm). All chemicals were purchased from Sigma-Aldrich (St. Louis, MO, USA) and used without further purification. Organic solvents were purchased from Sinopharm chemical reagent Co., Ltd and purified by distillation and moisture was excluded from the glass apparatus using CaCl_2_ drying tubes.

### Preparation of SWCNTs-MG

Purified SVCV G protein was prepared according to our previous study [24]. Modification of SWCNTs-MG was prepared by the chemical combination. Briefly, the functionalized mannose solution (100 mg/mL) was added into 500 mL G protein solution dissolved in boric acid solution and then stirred for 24 h at room temperature. After the resulting mixture was filtered and washed thoroughly with PBS (pH 7.4), the mannosylated G protein (MG) was obtained. Conjugation of MG with o-SWCNTs to form SWCNTs-MG was according to previous studies. Conjugation of G/MG/SWCNTs-G/SWCNTs-MG with fluorescein isothiocyanate (FITC) was according to previous study [28].

The SWCNTs-MG was characterized by field emission scanning electron microscopy (FE-SEM, S-4800, Hitachi Ltd., Tokyo, Japan) and high resolution TEM (HR-TEM; Tecnnai G2 F20, USA). An X-ray photoelectron spectroscopy (XPS; PHI-5600, Russia) was used to analyze elemental compositions of o-SWCNTs, SWCNTs-G and SWCNTs-MG. Theromogravimetric analysis (TGA; Mettler Toledo, Switzerland) was carried out to further qualitatively or quatitatively characterize the modification of o-SWCNTs, SWCNTs-G and SWCNTs-MG. Particle size (nm) and zeta potential (mV) of o-SWCNTs, SWCNTs-G and SWCNTs-MG were determined by dynamic light scattering (DLS) analysis (ZEN3600, Malvern, UK).

### Safety evaluation of SWCNTs-MG

For the cytotoxicity of nanovaccine was determined by the 3-(4,5-dimethyl-2-thiazolyl)-2,5-diphenyl-2H-tetrazolium bromide (MTT) assay (Sigma, USA) following the standard protocol.

For the safety evaluation in vivo, 60 carps were bathed in SWCNTs-MG vaccine at 60 mg/L for 24 h. 10 treated carp and 10 normal carp were randomly selected to isolate tissues and organs (brain, gill, intestinal tract, kidney, liver, and spleen), then tissue sections were made and stained. The remaining carp were transferred to clean water for normal feeding. The health and survival of the fish were observed for 60 days.

### Cellular and tissular uptake of nanovaccine

In the cell uptake study, G, MG, SWCNTs-G, and SWCNTs-MG were incubated with 6 × 10^7^ macrophage for 24 h. Macrophages were obtained by centrifugation at 1500 rpm for 2 min. Furthermore, treated macrophages were immunostained with the tissue resident macrophage marker F4/80 primary antibody (1:250, Abcam, Cambridge, England), Cy3-labeled secondary antibody (1:1000, Beyotime. China), and DAPI (Beyotime. China). The cell uptake of fluorescently labeled nanovaccine was analyzed by BD FACSAria flow cytometry (BD, USA) and confocal microscopy (Leica, Germany).

For the detection of nanovaccine in vaccinated fish, carps (80 tail in total) were randomly divided into 4 groups (20 tail/group): G, MG, SWCNTs-G, and SWCNTs-MG group. Each group was immersed with G-FITC, MG-FITC, SWCNTs-G-FITC, and SWCNTs-MG-FITC at 30 mg/L for 6 h, respectively. After vaccination, fishes were transferred to clean water. Tissues including muscle, gill, intestine, kidney, spleen, and liver were isolated from vaccinated fish. Then tissues sections were made and then observed in confocal microscopy (Leica, Germany). Image J software was used to quantify the intensity of fluorescence in each group.

### In vivo fluorescence imaging

Carps (n = 60) were randomly selected and treated with FITC-labeled SWCNTs-MG at a concentration of 30 mg/L for 6 h. After 6 h, the fish were transferred to clean water for breeding. tissues (gills, kidneys, spleen, liver, anterior intestine, middle intestine, and posterior intestine) were isolated from the vaccinated carps. Living body imaging system AniView 100 (BLT, China) was used to observe vaccinated carps and tissues at 5 different time points (0, 0.1, 6, 12, and 24 h) after the immersion immunization.

### Analysis on the activation and antigen presentation of APCs in vivo

Carps were randomly divided into 7 groups (30 fish per group) and then immersed with PBS, SWCNTs, Mannose, G, MG, SWCNTs-G, and SWCNTs-MG at a concentration of 30 mg/L for 6 h, respectively. After the bath administration, these fish were transferred to clean water for breeding. At 7 day after the immersion, head kidney tissues were isolated from vaccinated carps in different groups (n = 9, per group), then those tissues were homogenized, centrifugated and stored at − 80 °C. The activation and antigen presentation of APCs were analyzed by using fish CD4, MHC-II, and CD80/86 ELISA kits (Renjiebio, China), respectively.

### Immune response analysis

ELISA was used to analyze the antibody response and enzyme activity in vaccinated fish. The titers of the antibodies were measured by ELISA (Enzyme-linked immunosorbent assay) as described elsewhere [32]. For analyses of the presence of specific, neutralizing antibodies, vaccinated and control fish (3 fish of 3 independent experiments) were sampled weekly until 6 weeks for antibody determination. Serum samples preparation and determination were according to previous method. Briefly, the blood collected from the caudal vein of common carp was placed overnight at 4 °C and then centrifugated at 5000*g* for 15 min. The supernatant was collected and stored at − 20 °C until use. Purified recombinant M protein was used as antigen. Anti-Common carp (*Cyprinus carpio carpio*)/Koi carp (*Cyprinus carpio koi*) IgM monoclonal antibody labeled with horseradish peroxidase (Aquatic Diagostics Ltd., England) was diluted with PBS containing 3% skimmed milk at the ratio of 1:1000 before use, followed by color development using tetramethylbenzidine, TMB (Tiangen Biotech, Beijing, China) was used as colorimetric substrate. The plate was read at 450 nm by using a precision microplate reader (Molecular Devices Corp., Palo Alto, CA).

For RNA isolation and cDNA synthesis cDNA synthesis, total RNAs were obtained from the kidney tissues in each group (3 fish per group) at 1, 3, 7, 14 and 21 days after vaccination with TRIzol reagent. HiScript Q Select RT SuperMix for aPCR (+gDNA wiper) (Vazyme, China) was performed to reverse transcribed the purified RNA into cDNA.

Quantitative real-time PCR (qRT-PCR) was performed with CFX96 Real-Time PCR Detection System (Bio-Rad, USA) using AceQ^®^ qPCR SYBR^®^ Green Master Mix (Vazyme, China) with the following procedure: 95 °C for 5 min and 40 cycles at 95 °C denaturation for 15 s, followed by 60 °C annealing for 60 s. The extracted DNA were used as template for RT-PCR amplification with specific primers SM-F/R. The *β*-*actin* was used as an internal control (Additional file 1: Table S1). All qRT-PCR reactions were performed for three biological replicates and repeated with two independent samples. Relative mRNA expression was calculated using 2^−△△Ct^ method with the formula, F = 2^−ΔΔCt^, ΔΔCt = (Ct, target gene − Ct, reference gene) − (Ct, target gene − Ct, reference gene) control.

### Statistical analysis

All data were analyzed using SPSS Software 21 (IBM). Differences in cell and tissues uptake capability were analyzed with Student’s *t* test (*P < 0.05, **P < 0.01); Differences in APCs activation, antibody production and enzyme activities were analyzed by Duncan’s test, values with different letters are significant (*P* < 0.05); The relative percentage survival was analyzed with Log-rank (Mantel-Cox) Test (*P < 0.05, **P < 0.01).

## Supplementary information

**Additional file 1: Table S1.** Primers used for the analysis of mRNA expression displayed; **Figure S1.** Synthetic route of mannose showed. **Figure S2.**^1^HNMR and ^13^C NMR spectra of modified mannose showed.

## Data Availability

All data generated or analyzed during this study are included in this published article.
