# Supplementary material for: Application of antigen presenting cell-targeted nanovaccine delivery system in rhabdovirus disease prophylactics using fish as a model organism
Source: J Nanobiotechnology. 2020 Jan 30;18:24. doi: 10.1186/s12951-020-0584-x (PMC6993333; doi:10.1186/s12951-020-0584-x)
Supplement: Supplementary file 1 — Additional file 1: Table S1. Primers used for the analysis of mRNA expression displayed; Figure S1. Synthetic route of mannose showed. Figure S2.1HNMR and 13C NMR spectra of modified mannose showed. [file 12951_2020_584_MOESM1_ESM.docx]

Additional Information

**Application of antigen presenting cell-targeted nanovaccine delivery system in rhabdovirus disease prophylactics**

Chen Zhang, Gao-Xue Wang, Bin Zhu*

College of Animal Science and Technology, Northwest A&F University, Yangling 712100, China

*Corresponding author:

Bin Zhu

Northwest A&F University, Xinong Road 22nd, Yangling, Shaanxi 712100, China.

Tel./fax: +86 29 87092102.

E-mail address: zhubin1227@126.com (B. Zhu).

**Table S1.** Primers used for the analysis of mRNA expression by qRT-PCR.

| Genes | Accession no. | | Primer sequences (from 5’ to 3’) | Product size (bp) |
| --- | --- | --- | --- | --- |
| *β-actin* | M24113 | Forward | GCTATGTGGCTCTTGACTTCG | 85 |
|  |  | Reverse | CCGTCAGGCAGCTCATAGCT | |
| *TNF-α* | AJ311800.2 | Forward | TGTGCCGCCGCTGTCTGCTTCACGCT | 291 |
|  |  | Reverse | GATGAGGAAAGACACCTGGCTGTAGA | |
| *IL-10* | JX524550.1 | Forward | GTCATCCTTTCTGCTCTGGTT | 91 |
|  |  | Reverse | CCACAAATGAGCAACAGTCA | |
| *Cxcr 1* | AB010468.1 | Forward | GCAAATTGGTTAGCCTGGTGA | 144 |
|  |  | Reverse | AGGCGACTCCACTGCACAA | |
| *Cxca* | AJ421443 | Forward | CTGGGATTCCTGACCATTGGT | 88 |
|  |  | Reverse | GTTGGCTCTCTGTTTCAATGCA | |
| *IFNg2b* | JX181980.1 | Forward | GCTCAAGAAGTATGCAGAAACTC | 151 |
|  |  | Reverse | TCTGGCTTGTCGTCTCCT | |
| *IgM* | AB004105 | Forward | CACAAGGCGGGAAATGAAGA | 145 |
|  |  | Reverse | CTGATAAAGCTTTGCACTTCAGCA | |
| *CD4* | DQ400124.1 | Forward | AGTGGGATCCAAAGGGCGAA | 214 |
|  |  | Reverse | ATTCCAGAGACAGAGAGT | |
| *MHC-*Ⅱ | S62611.1 | Forward | TGCAGTGCCTATGACTTC | 191 |
|  |  | Reverse | GAGCTGGCGTGCTCCA | |


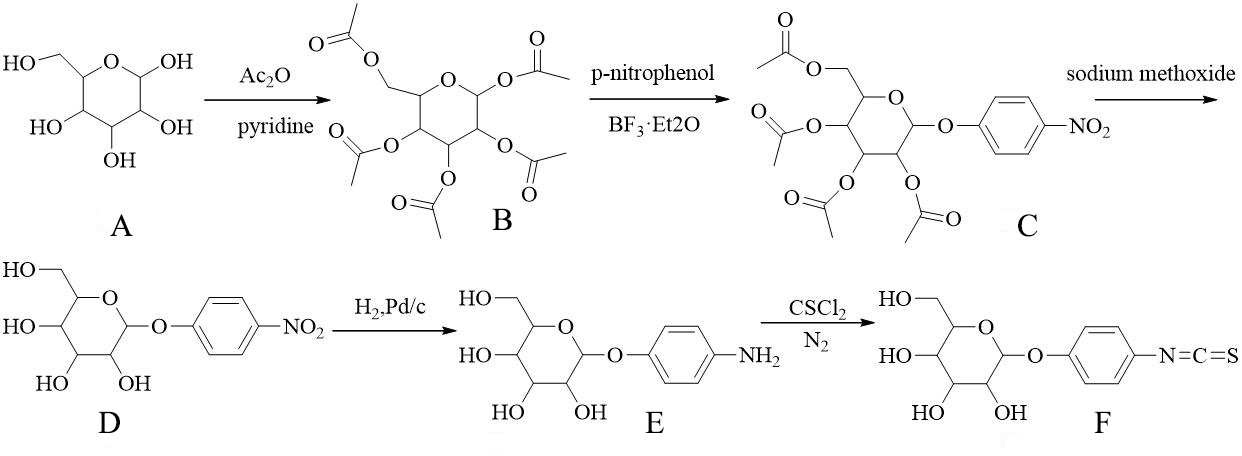


**Figure S1.** Synthetic route of mannose. (A) D-mannose; (B) acetyl mannose; (C) p-nitrophenol-tetraoxy acetyl-d-pyranomannan; (D) p-nitrophenol-d-pyranomannan; (E) p-aminophenol-d-pyranomannan; (F) 1-(Isothiocyanates phenol)-2,3,4,6-O-α-D-mannose.


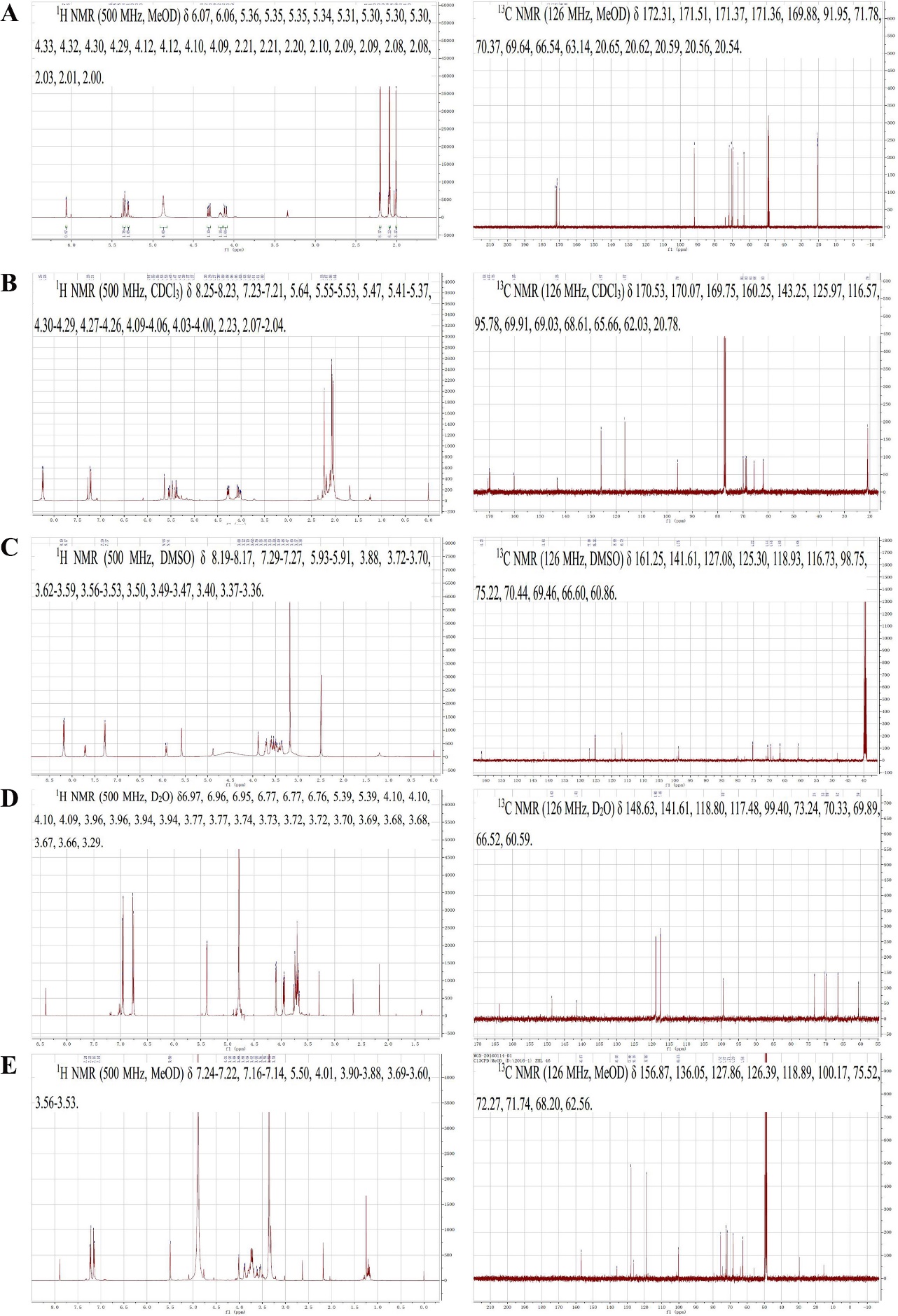


**Figure S2.** ^1^HNMR and ^13^C NMR spectra of modified mannose: (A) D-mannose; (B) acetyl mannose; (C) p-nitrophenol-tetraoxy acetyl-d-pyranomannan; (D) p-nitrophenol-d-pyranomannan; (E) p-aminophenol-d-pyranomannan; (F) isothiocyanate phenol-pyranomannan**.**
